# Multimodal Physiotherapy Following Patellar Stabilization Surgery in Five Small‐Breed Dogs: A Case Series

**DOI:** 10.1002/vms3.70900

**Published:** 2026-03-30

**Authors:** Majid Rajabian, Mahdi Esmaeeli, Kazem Malmir, Arman Abdous

**Affiliations:** ^1^ Department of Physiotherapy, School of Rehabilitation Tehran University of Medical Sciences Tehran Iran; ^2^ Department of Clinical Sciences, Faculty of Veterinary Medicine, Karaj Branch Islamic Azad University Karaj Iran

**Keywords:** canine rehabilitation, medial patellar luxation, physiotherapy, postoperative recovery, small‐breed dogs

## Abstract

Medial patellar luxation (MPL) is a common orthopaedic disorder in small‐breed dogs (*Canis lupus familiaris*) and frequently requires surgical stabilization to restore normal joint alignment. However, postoperative complications such as residual lameness, quadriceps muscle atrophy and reduced stifle joint mobility may delay functional recovery. This case series describes the clinical outcomes associated with a structured multimodal physiotherapy protocol implemented following MPL stabilization surgery in five small‐breed dogs diagnosed with Grade III–IV MPL. After surgical correction, all dogs underwent a standardized 4‐week rehabilitation programme consisting of neuromuscular electrical stimulation, therapeutic ultrasound, manual therapy and targeted functional exercises, with a total of 12 treatment sessions. Functional recovery was assessed using quadriceps muscle girth, passive stifle extension, lameness scoring and the Timed Up and Go (TUG) test. Evaluations were performed at baseline prior to physiotherapy, immediately after completion of the 4‐week rehabilitation programme, and at a 1‐month follow‐up. All dogs demonstrated progressive improvements in muscle girth, joint extension and functional mobility, accompanied by consistent reductions in lameness scores. Improvements observed following rehabilitation were maintained at the 1‐month follow‐up evaluation. These findings suggest that early integration of a structured multimodal physiotherapy protocol may enhance postoperative functional recovery and mobility in small‐breed dogs undergoing surgical stabilization for MPL.

## Graphical abstract

Five small‐breed dogs undergoing surgery for medial patellar luxation received a structured 4‐week multimodal physiotherapy programme. Rehabilitation improved quadriceps muscle mass, stifle joint mobility, gait function and reduced lameness. Benefits were maintained at 1‐month follow‐up.

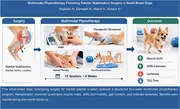

## 1 Introduction

Medial patellar luxation (MPL) is among the most frequently diagnosed orthopaedic disorders in dogs, particularly in small‐breed companion animals, and represents a major cause of hindlimb lameness, reduced mobility and impaired quality of life (Nunamaker and Blauner 1985; Linney et al. 2011). Of the recognized forms including medial, lateral or bilateral luxation, medial displacement predominates and is strongly associated with congenital skeletal malalignment, soft tissue contracture or traumatic disruption of stabilizing structures (Hayes et al. 1994; Arthurs and Langley‐Hobbs 2006). Breed predisposition is well established, with Chihuahuas, Shih Tzus, Pomeranians and Yorkshire Terriers consistently overrepresented in clinical cohorts (Priester 1972; LaFond et al. 2002).

The severity of MPL is categorized into four grades, with Grades III and IV often requiring surgical stabilization due to irreducible luxation or associated skeletal deformities (Roush 1993; Akaraphutiporn et al. 2024). Surgical procedures, including trochlear wedge recession, tibial tuberosity transposition and soft tissue balancing, aim to realign the extensor mechanism and restore patellar stability. Nevertheless, even after technically successful surgery, functional deficits frequently persist, such as lameness, quadriceps atrophy, reduced passive range of motion (PROM) and proprioceptive dysfunction, which may delay or compromise recovery (Millis and Levine 2013).

In recent years, postoperative rehabilitation has gained increasing recognition as an important adjunct to orthopaedic surgery in veterinary medicine. Early and structured physiotherapy may reduce periarticular fibrosis, counteract disuse muscle atrophy, restore joint mobility and improve neuromuscular coordination (Lopez de la oliva Cases and Grierson 2019; Mahaseth and Raghul 2021). Commonly applied techniques include neuromuscular electrical stimulation (NMES), therapeutic ultrasound (US), manual therapy and targeted exercise programmes incorporating proprioceptive and strengthening activities (Millis and Ciuperca 2015; Karasiewicz et al. 2022).

Despite this progress, systematic evaluation of standardized multimodal physiotherapy protocols following MPL surgery in small‐breed dogs remains limited. Published reports are predominantly single‐case descriptions or lack consistent and quantifiable functional outcome measures such as PROM, standardized lameness scoring, muscle girth assessment and the Timed Up and Go (TUG) test (Padilha Filho et al. 2005; Frye et al. 2022). Variability in referral patterns, access to rehabilitation services and the absence of routinely applied objective assessment tools may have contributed to the limited availability of comparable multi‐case data.

This case series describes the outcomes of five small‐breed dogs undergoing structured rehabilitation following surgical correction of Grade III or IV MPL. The objective was to evaluate a standardized multimodal physiotherapy protocol including NMES, US, manual therapy and targeted functional exercises using defined and reproducible recovery parameters. This report contributes to the limited multi‐case veterinary literature on structured postoperative rehabilitation with objective outcome assessment in small‐breed dogs following MPL stabilization.

## 2 Case History

All five dogs in this case series were small‐breed patients (≤ 10 kg) diagnosed with Grade III or IV MPL, confirmed by orthopaedic examination and diagnostic radiography. Each underwent surgical correction via trochlear wedge recession, with adjunctive procedures such as tibial tuberosity transposition or lateral retinacular imbrication performed where indicated. Postoperative recovery was uncomplicated, and all dogs were referred for physiotherapy within 10–21 days of surgery, once surgical incisions had healed and ambulation had stabilized.

A standardized multimodal physiotherapy protocol was implemented across all cases, consisting of 12 sessions delivered over 4 weeks (three sessions per week). Each session began with NMES applied to the quadriceps muscle group using adhesive electrodes positioned over the vastus lateralis and rectus femoris. Parameters included a frequency of 35 Hz, pulse duration of 250 µs and a 1:2 on/off ratio, administered for 10 min to achieve visible muscle contraction. This was followed by the US applied to the hamstring region at 1 MHz frequency and 1.2 W/cm^2^ intensity, in continuous mode for 5 min, using slow circular movements to enhance tissue extensibility.

Manual therapy techniques, including effleurage and kneading of the quadriceps and hamstrings, were applied for approximately five min to improve circulation and reduce muscular tension (Formenton et al. 2017). PROM exercises consisted of 20 repetitions of controlled stifle flexion and extension performed with the patient in lateral recumbency. Active range of motion (AROM) and functional training were then introduced, including sit‐to‐stand transitions and walking over uneven foam pads to encourage symmetrical weight‐bearing and re‐establish neuromuscular control. Proprioceptive and strengthening exercises were progressively integrated as tolerated, involving standing on wobble cushions, balancing on Swiss balls or rocker boards and walking on inclined ramps to challenge dynamic stability.

Assessments were conducted at three time points: baseline (pre‐treatment), immediately after completion of the 4‐week rehabilitation programme (Week 4) and at a 1‐month post‐treatment follow‐up (Week 8). Outcome measures included mid‐thigh quadriceps girth (measured with a spring‐tension tape at 70% of the distance between the greater trochanter and patella in lateral recumbency), passive stifle extension angle (measured with a goniometer aligned to the femoral and fibular axes), functional mobility via the TUG test (transition from sternal recumbency to standing, followed by walking 10 body lengths) and lameness using a 6‐point scale (0 = normal, 5 = non‐weight bearing). All assessments were performed by the same physiotherapist under veterinary supervision to ensure consistency.

**Case 1**: An 8‐year‐old female Spitz (10 kg) with Grade IV left MPL was presented with pronounced quadriceps atrophy and limited stifle extension. Physiotherapy began 2 weeks postoperatively. Progressive gains in muscle girth, extension range and TUG performance were observed throughout therapy and maintained at the 1‐month post‐treatment follow‐up. Lameness improved steadily to near‐complete resolution.

**Case 2**: A 6‐year‐old male Pomeranian (4.5 kg) with Grade III right MPL underwent wedge recession with tibial tuberosity transposition. Physiotherapy commenced 10 days post‐surgery, resulting in measurable improvements in quadriceps girth, joint mobility and gait efficiency. Lameness resolved fully by the end of therapy and remained absent at the 1‐month post‐treatment follow‐up.

**Case 3**: A 10‐year‐old female Chihuahua (3.2 kg) with Grade IV left MPL began physiotherapy 3 weeks post‐surgery. Initial assessments revealed severe limitations in both flexion and extension and delayed functional recovery. Despite the delayed start, rehabilitation yielded substantial gains in PROM, gait and lameness scores by Week 8.

**Case 4**: A 7‐year‐old male Miniature Pinscher (5.1 kg) with bilateral Grade III MPL underwent staged surgical correction. Physiotherapy began 2 weeks after the second surgery. Bilateral improvements in quadriceps girth, stifle extension and dynamic balance were recorded, accompanied by a full return to weight‐bearing and normal gait.

**Case 5**: A 9‐year‐old female Shih Tzu (6.8 kg) with Grade IV right MPL underwent wedge recession and lateral retinacular imbrication. She began rehabilitation with moderate pain and mobility restriction. With early analgesic support and consistent physiotherapy, she achieved restoration of joint mobility and functional independence by programme completion.

**FIGURE 1:**
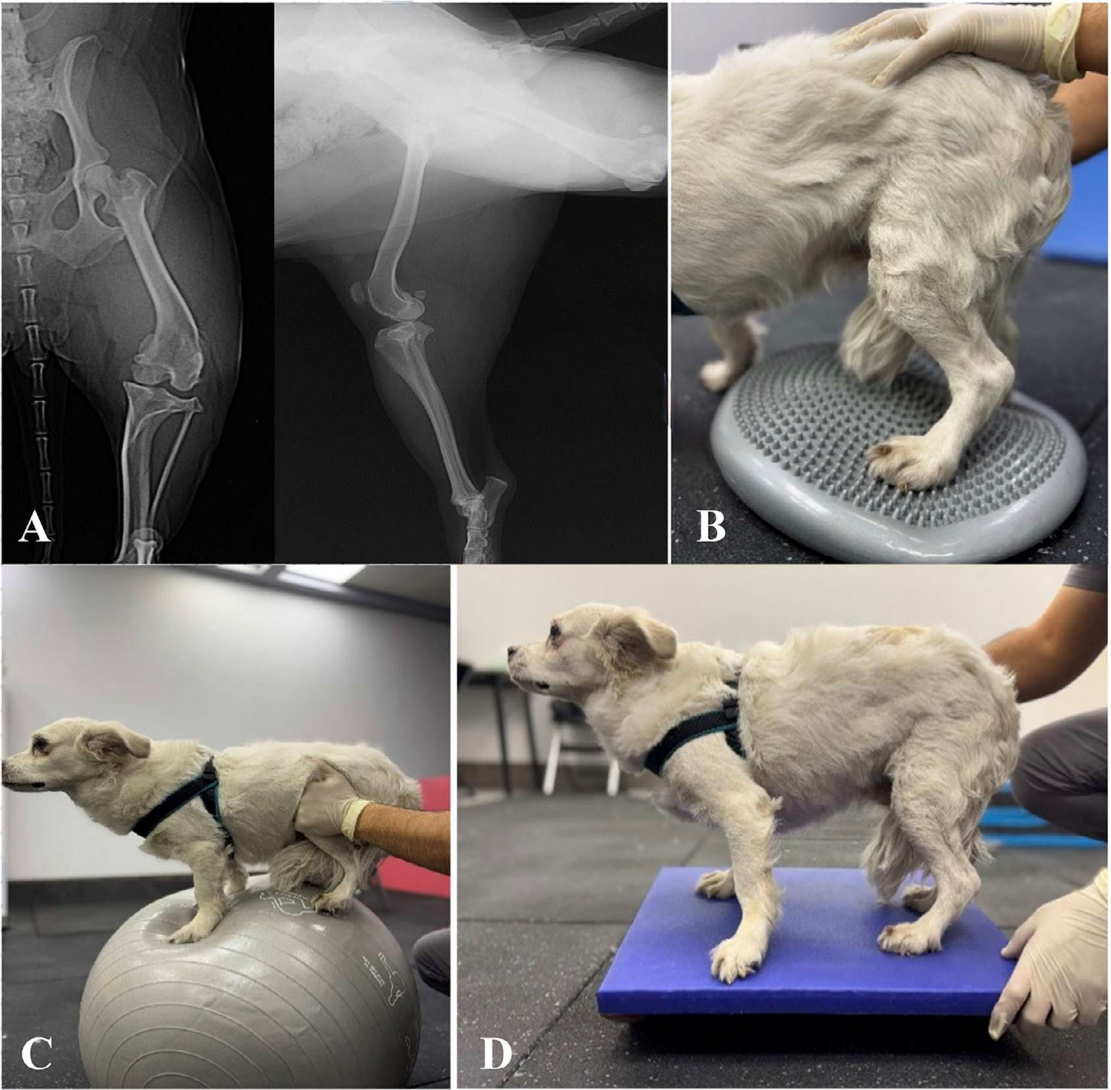
(A) Craniocaudal and mediolateral radiographic views of the left hindlimb pre‐surgery. (B) Dog standing on wobble cushion. (C) Dog performing Swiss ball balancing exercise. (D) Standing posture on rocker board during proprioceptive training.

**FIGURE 2:**
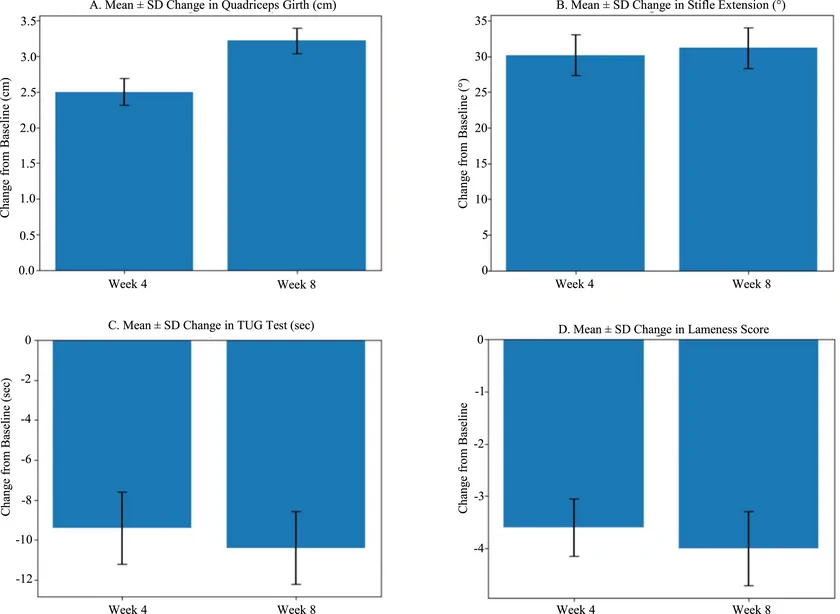
Mean ± SD change from baseline in functional and clinical outcomes following multimodal physiotherapy. Bar graphs illustrate the mean ± standard deviation (SD) change from baseline in (A) quadriceps girth, (B) stifle extension angle, (C) Timed Up and Go (TUG) test performance and (D) lameness score at post‐treatment (Week 4) and at the 1‐month post‐treatment follow‐up (Week 8) in five small‐breed dogs undergoing rehabilitation after medial patellar luxation surgery. Positive values indicate improvement for quadriceps girth and stifle extension, whereas negative values indicate improvement for TUG time and lameness score.

Figure 1 illustrates the clinical procedures, Table 1 presents individual patient data across time points and Figure 2 summarizes the mean ± SD change from baseline for each outcome measure at Week 4 and Week 8.

**TABLE 1:** Outcome measures across five dogs undergoing multimodal physiotherapy after MPL surgery.

Case	Time point	Quadriceps girth (cm)	Stifle extension (°)	TUG test (sec)	Lameness score^a^
1	Baseline	14.5	136	16	4
	Post‐treatment (Week 4)	16.8	166	9	1
	1‐month post‐treatment follow‐up (Week 8)	17.5	167	8	0
2	Baseline	13.2	138	17	3
	Post‐treatment (Week 4)	15.7	166	8	0
	1‐month post‐treatment follow‐up (Week 8)	16.4	167	7	0
3	Baseline	12.0	130	22	5
	Post‐treatment (Week 4)	14.4	164	10	1
	1‐month post‐treatment follow‐up (Week 8)	15.2	165	9	0
4	Baseline	14.8	140	18	4
	Post‐treatment (Week 4)	17.3	167	9	0
	1‐month post‐treatment follow‐up (Week 8)	18.0	168	8	0
5	Baseline	13.5	135	19	4
	Post‐treatment (Week 4)	16.3	167	9	0
	1‐month post‐treatment follow‐up (Week 8)	17.0	168	8	0

Abbreviations: MPL, medial patellar luxation; TUG, Timed Up and Go.

^a^Scored from 0 to 5, where 0 = no lameness and 5 = non‐weight bearing.

## 3 Discussion

This case series highlights the clinical importance of structured, multimodal physiotherapy in promoting postoperative recovery following MPL surgery in small‐breed dogs. Across all five patients, progressive improvements were consistently observed in PROM, quadriceps muscle girth, lameness scores and functional mobility as assessed by the TUG test. These findings reinforce prior evidence supporting the role of early rehabilitation in restoring joint function, reducing pain and enhancing gait performance (Millis and Levine 2013; Mahaseth and Raghul 2021).

The observed increases in stifle extension angles suggest reduced periarticular stiffness and capsular fibrosis, likely attributable to passive stretching, manual therapy and neuromuscular reactivation. Similar improvements in joint kinematics have been documented in canine orthopaedic rehabilitation, underscoring the value of PROM and mobilization techniques in postoperative care (Padilha Filho et al. 2005). Gains in quadriceps circumference reflect reversal of disuse atrophy and successful hypertrophy through NMES and progressive strengthening exercises. These outcomes are consistent with previous reports describing the use of NMES in canine rehabilitation (Karasiewicz et al. 2022; Frye et al. 2022).

Functional recovery, as demonstrated by improved TUG performance, highlights the benefits of proprioceptive training, balance exercises and gait retraining strategies. These interventions may be particularly critical for small‐breed patients, in whom postural control and core stability are often compromised following surgery (Millis and Ciuperca 2015). The slower recovery noted in Case 3, the oldest dog and the one referred later postoperatively, supports previous findings that age‐related sarcopenia and delayed initiation of therapy are associated with slower improvement (Lopez de la oliva Cases and Grierson 2019). This observation underscores the importance of early referral for physiotherapy to maximize functional gains.

Although encouraging, these results must be interpreted with caution. Limitations include the small sample size, the absence of a control group and the lack of advanced objective measures such as force plate gait analysis or validated pain‐scoring instruments, including the Canine Brief Pain Inventory. Consequently, improvements cannot be attributed solely to the physiotherapy protocol, as natural healing and placebo effects may have contributed (Arthurs and Langley‐Hobbs 2006). Nonetheless, the structured design of the protocol, standardized implementation and consistent improvements across multiple outcome domains strengthen the clinical relevance of these findings.

To our knowledge, this report adds to the limited multi‐case veterinary literature describing structured multimodal rehabilitation with quantifiable functional outcomes in small‐breed dogs following MPL stabilization. Previous publications have predominantly consisted of single‐case descriptions or reports lacking standardized and objective assessment of recovery parameters. By incorporating accessible and reproducible clinical measures such as PROM, muscle girth assessment, lameness scoring and the TUG test, this series provides a practical framework for structured postoperative rehabilitation in routine veterinary practice.

Future research should aim to confirm these findings in larger, controlled cohorts and to integrate objective biomechanical and pain assessment tools. Protocols tailored to patient‐specific variables such as age, morphology and surgical technique may further refine outcomes. Ultimately, structured multimodal physiotherapy holds strong potential to accelerate recovery, reduce complications and improve long‐term quality of life in dogs undergoing MPL surgery.

## 4 Conclusion

This case series demonstrates the clinical value of a structured multimodal physiotherapy protocol in supporting recovery after patellar stabilization surgery in small‐breed dogs. Consistent improvements were observed in muscle mass, joint mobility, lameness scores and functional mobility across all patients. These findings contribute to the growing body of evidence supporting standardized postoperative rehabilitation with objective outcome assessment in this population. Early and structured physiotherapy appears to represent a practical and reproducible strategy to enhance recovery, improve prognosis and promote functional independence following orthopaedic intervention.

## Author Contributions

Conceptualization: Majid Rajabian. Methodology: Majid Rajabian. Investigation: Kazem Malmir and Mahdi Esmaeeli. Formal analysis: Kazem Malmir and Mahdi Esmaeeli. Supervision: Majid Rajabian and Mahdi Esmaeeli. Project administration: Arman Abdous. Resources: Arman Abdous. Writing – original draft: Majid Rajabian, Mahdi Esmaeeli and Arman Abdous. Writing – review and editing: All authors.

## Funding

The authors have nothing to report.

## Ethics Statement

The authors confirm that the ethical policies of *Veterinary Medicine and Science*, as noted on the journal's author guidelines page, have been adhered to. The study was approved by the Ethics Review Committee of Tehran Veterinary Hospital, Tehran, Iran (Approval Code: VH2024‐IR‐08). All procedures were performed in accordance with international standards for the humane treatment of animals. Informed consent was obtained from the owners of all participating animals.

## Conflicts of Interest

The authors declare no conflicts of interest.

## Data Availability

All data generated or analysed during this study are included in this published article. Further inquiries can be directed to the corresponding author.
